# Identifying improvements for delivery room resuscitation management: results from a multicenter safety audit

**DOI:** 10.1186/s40748-014-0006-x

**Published:** 2015-01-22

**Authors:** Erika M Edwards, Roger F Soll, Karla Ferrelli, Kate A Morrow, Gautham Suresh, Joanna Celenza, Jeffrey D Horbar

**Affiliations:** Vermont Oxford Network, Burlington, VT USA; Department of Mathematics and Statistics, University of Vermont, Burlington, VT USA; Department of Pediatrics, University of Vermont, Burlington, VT USA; Department of Pediatrics, Dartmouth Hitchcock Medical Center, Lebanon, NH USA

**Keywords:** Cooperative behavior, Delivery rooms, Infant, Newborn, Premature, Patient care team/organization & administration, Resuscitation

## Abstract

**Background:**

Stabilization and resuscitation of a newborn infant is a complex activity that involves multiple team members. Neonatal intensive care units (NICU) participating in the Vermont Oxford Network (VON) iNICQ 2012 quality improvement collaborative reported on delivery room care policies and guidelines and submitted information on up to 10 consecutive deliveries attended by NICU team members. Teams received immediate feedback on their local performance and a summary of results from all participating units for use in quality improvement planning.

**Results:**

Most of the 84 NICU teams that participated in the audit had policies or guidelines about which deliveries required NICU team attendance (83%), personnel who should attend (81%), and their required training (79%). Fewer had policies about briefing prior to the delivery (8%), debriefing after delivery (6%), or communicating with family members (10%). Eighty-one percent of NICUs reported using simulation-based resuscitation training, 14% used a safety checklist, and 2% videotaped deliveries for review. Of the 609 audited deliveries, 88% had team member attendance that conformed to unit policy, 66% had a briefing before delivery, 19% had a debriefing after delivery, and 92% had family communication occur within 30 minutes.

**Conclusions:**

NICU teams can improve the quality and safety of delivery room care by implementing formal tools designed to facilitate teamwork such as briefings, debriefings, checklists, and videotape reviews. Rapid online audits are effective methods for helping teams identify opportunities for improvement.

**Electronic supplementary material:**

The online version of this article (doi:10.1186/s40748-014-0006-x) contains supplementary material, which is available to authorized users.

## Background

An estimated 10 percent of all newborns require assistance breathing after birth, with one percent of infants needing more complicated resuscitation measures [1]. In particular, very low birth weight infants, those weighing less than 1500 grams at birth, are more likely to require intubation, chest compressions, or administration of medications during initial resuscitation [2] and subsequent admission to a neonatal intensive care unit (NICU). High-quality care during resuscitation lays the foundation for a successful transition to the NICU and extrauterine life.

Delivery room resuscitation and stabilization is a complex activity involving multiple health care professionals from different disciplines [3]. Effective teamwork is essential to achieve optimal outcomes in health care [4-9]. A call to action for quality patient care in labor and delivery, endorsed by seven influential professional organizations, specifically recommends training clinicians in the principles of teamwork and shared decision-making to improve outcomes [10]. Thomas and colleagues identified optimal teamwork behaviors for neonatal resuscitation including sharing information, evaluating plans, and prioritizing and distributing the workload among team members [11]. Teaching team behaviors in conjunction with a skills-based curriculum such as the Neonatal Resuscitation Program (NRP; [12]) can significantly improve teamwork [13-18] and quality of care [13,15]. To facilitate effective teamwork, the 2010 International Consensus Guidelines on Neonatal Resuscitation recommend briefing and debriefing, the process of reviewing and communicating pertinent facts about the resuscitation before and after events [19]. The most recent revision of the NRP added simulation-based education and post-scenario debriefing to help trainees practice teamwork skills [12].

An additional important step to improving team behaviors is systematic documentation and evaluation of standard teamwork practices [9]. To function effectively, team members need to know who should be present at a neonatal resuscitation, each team member’s roles and responsibilities, and the skills required to fulfill those roles [9]. To reinforce optimal teamwork behaviors, briefing and debriefing should be a standard part of every resuscitation procedure. Documenting such policies and guidelines reinforces organizational support for quality care and emphasizes a culture of safety [4,5,9].

As part of ongoing efforts to help patient care teams improve the quality and safety of medical care for newborn infants and their families, Vermont Oxford Network, a non-profit voluntary collaboration of health care professionals established in 1988 [20], coordinated an online VON Days quality audit of guidelines and practices for teamwork during delivery room resuscitation. The quality audit was part of iNICQ 2012, an Internet-based improvement collaborative in which multidisciplinary teams from around the world worked together under the guidance of expert faculty to make measurable improvements in the quality and safety of respiratory care. A key component of this collaborative, as with other face-to-face collaboratives organized by VON, was the use of potentially better practices (PBPs), practices derived from the best evidence available, whose implementation through Plan-Do-Study-Act cycles is likely to improve the processes or outcomes of interest. PBPs are improvement ideas with logical appeal and practical application that can be implemented, tested, and measured at the local level after modification for local context [21-23]. We call these evidence-based practices “potentially better” rather than “better” or “best” because until the practices are evaluated, customized, and tested at the local level, teams will not know if they are truly better.

iNICQ faculty identified three potentially better practices (PBPs) for delivery room resuscitation and respiratory care of very low birth weight infants during the first hour of life: teamwork and communication in the delivery room, use of a team approach to respiratory care during the first hour of life, and maintenance of normal temperature in very low birth weight infants. Over the course of the 2012 iNICQ, clinical and quality improvement expert faculty conducted five web-based sessions. Before each iNICQ session, teams completed a VON Day Audit to assess local practices.

The VON Day Audit for resuscitation focused on the first PBP, improving teamwork and communication in the delivery room. Of 144 centers eligible for this audit, 84 centers participated (Additional file 1). The audit asked about guidelines or policies at the unit level and whether they were applied at the infant level. Unit level policies or guidelines included: which deliveries required attendance by an individual or team responsible for the infant after delivery; required personnel; required training; pre-delivery briefings; debriefings; and family communication after delivery. Data collectors reported whether resuscitation teams attending deliveries routinely used checklists to ensure the quality and safety of the deliveries, whether the unit had a program for simulation-based neonatal resuscitation training, and whether the unit routinely videotaped deliveries/resuscitations of high-risk infants.

Each unit was asked to evaluate up to ten deliveries of subsequent NICU admissions from February 6 to 17, 2012. Eligible infants were those stabilized or resuscitated in the delivery room, defined as the place where the preponderance of stabilization and resuscitation occurred, and subsequently admitted to the neonatal intensive care unit. For each delivery, auditors reported the infant’s gestational age at birth and delivery characteristics, how many members of the resuscitation team attended the delivery, who attended, whether the composition of the team met unit policies or guidelines, whether the team performed a briefing, a debriefing within 24 hours of delivery, and whether a member or representative of the resuscitation team spoke to the mother or other family member within 30 minutes of initial resuscitation. Units could answer “family member or parent not available” if the mother was under general anesthesia and it was known that no other family member authorized to receive medical information about the infant was in the hospital within 30 minutes of the delivery. If the delivery was attended by a single individual responsible for the initial resuscitation, a briefing was defined as a systematic communication prior to the delivery between the individual and one or more members of the obstetrical team to help prepare the individual for the delivery, and a debriefing was defined as a systematic communication within 24 hours after the delivery between the individual and one or more members of the NICU team responsible for monitoring the quality of delivery room practice. Delivery audits occurred either by direct observation in the delivery room or by interview within 24 hours after admission of infants requiring delivery room resuscitation.

Vermont Oxford Network provided a manual of operations and data collection forms. The audit employed a custom online data collection and reporting system built on the Microsoft ASP.NET application framework and integrated into the Vermont Oxford Network member website. Each unit received a confidential online report of results immediately following the audit and a summary report of all participating centers’ results shortly after audit completion.

For this analysis, measures relating to infant characteristics were reported within the context of the audited deliveries. Measures relating to NICU characteristics came from the last available annual Vermont Oxford Network member survey. Teaching hospitals were defined as those that had pediatrics residents, neonatal fellows, or other residents involved in care. Accordance with audit objectives was defined as the proportion of audited deliveries for which all four of the following were done: team composition met unit policy; briefing was performed; debriefing was performed; and family communication occurred immediately following delivery. Units without guidelines or policies were considered not to be in accordance with audit objectives. Deliveries for which a family member or parent was not available was considered to be in accordance. Only descriptive statistics are reported. All analyses were done in SAS 9.3 (Research Triangle Park, N.C.). The VON Day audit was approved by the institutional review boards of the University of Vermont and each participating hospital.

## Methods

### Participants

Vermont Oxford Network is a non-profit voluntary collaboration of health care professionals established in 1988 [20]. Member hospitals participating in iNICQ 2012, an interactive quality collaborative, participated in the VON Days Delivery Room Resuscitation Audit. Of 144 eligible centers, 84 centers participated (Additional file 1). Each unit was asked to evaluate up to ten deliveries of subsequent NICU admissions from February 6 to 17, 2012. The VON Day audit was approved by the institutional review boards of the University of Vermont and each participating hospital. Each unit designated one person responsible for data collection, who received a manual with standardized definitions and data collection forms.

### Measures

The audit asked about guidelines or policies at the unit level and whether they were applied at the infant level. Unit level policies or guidelines included: which deliveries required attendance by an individual or team responsible for the infant after delivery; required personnel; required training; pre-delivery briefings; debriefings; and family communication after delivery. Data collectors reported whether resuscitation teams attending deliveries routinely used checklists to ensure the quality and safety of the deliveries, whether the unit had a program for simulation-based neonatal resuscitation training, and whether the unit routinely videotaped deliveries/resuscitations of high-risk infants.

Eligible infants were those stabilized or resuscitated in the delivery room, defined as the place where the preponderance of stabilization and resuscitation occurred, and were subsequently admitted to the neonatal intensive care unit. Auditors completed individual patient data forms on up to 10 consecutive NICU admissions from the delivery room. For each delivery, auditors reported the infant’s gestational age at birth and delivery characteristics, how many members of the resuscitation team attended the delivery, who attended, whether the composition of the team met unit policies or guidelines, whether the team performed a briefing, a debriefing within 24 hours of delivery, and whether a member or representative of the resuscitation team spoke to the mother or other family member within 30 minutes of initial resuscitation. Units could answer “family member or parent not available” if the mother was under general anesthesia and it was known that no other family member authorized to receive medical information about the infant was in the hospital within 30 minutes of the delivery. If the delivery was attended by a single individual responsible for the initial resuscitation, a briefing was defined as a systematic communication prior to the delivery between the individual and one or more members of the obstetrical team to help prepare the individual for the delivery, and a debriefing was defined as a systematic communication within 24 hours after the delivery between the individual and one or more members of the NICU team responsible for monitoring the quality of delivery room practice.

### Data collection

Delivery audits occurred either by direct observation in the delivery room or by interview within 24 hours after admission of infants requiring delivery room resuscitation. Vermont Oxford Network provided a manual of operations and data collection forms. The audit employed a custom online data collection and reporting system built on the Microsoft ASP.NET application framework and integrated into the Vermont Oxford Network member website. Each unit received a confidential online report of results immediately following the audit and a summary report of all participating centers’ results shortly after audit completion.

Measures relating to infant characteristics were collected for the audited deliveries. Measures relating to NICU characteristics came from the last available annual Vermont Oxford Network member survey. Teaching hospitals were defined as those that had pediatrics residents, neonatal fellows, or other residents involved in care. Accordance with audit objectives was defined as the proportion of audited deliveries for which all four of the following were done: team composition met unit policy; briefing was performed; debriefing was performed; and family communication occurred immediately following delivery. Units without guidelines or policies were considered not to be in accordance with audit objectives. Deliveries for which a family member or parent was not available was considered to be in accordance. Only descriptive statistics are reported. All analyses were done in SAS 9.3 (Research Triangle Park, N.C.).

## Results

Of the 84 NICUs that participated in the audit (Additional file 1), 21% performed ventilation without restriction and major surgery, including cardiac surgery; 65% performed ventilation without restriction and major surgery except cardiac surgery; and 15% had restrictions on ventilation or surgery (Table 1). Most centers had policies or guidelines addressing which types of deliveries a resuscitation team needed to attend (83%), on the personnel who should attend such deliveries, and the training required by individuals who attend neonatal resuscitations (79%). Few units had policies regarding briefing prior to delivery (8%) or after delivery (6%), or communication between the teams or with families following delivery (10%). Of units, 81% reported using a simulation-based resuscitation program, 14% of units used a safety checklist, and only 2% videotaped deliveries for later review.

NICU teams audited 609 deliveries (median per hospital: 8; range, 1–10), of which 56% were done by direct observation. Infants were most likely to be 33 weeks gestational age or older (Table 2). Cesarean section (63%) and prematurity (61%) were the two reasons most frequently cited for the presence of a resuscitation team. NICU nurses (85%), respiratory therapists (63%), and neonatal nurse practitioners (51%) were highly likely to be part of the resuscitation teams. Of the deliveries, 88% met unit policy for team composition. Briefing occurred before 66% and debriefing occurred after 19% of audited deliveries. Communication with a family member occurred within 30 minutes of 92% of deliveries. Only 14% of audited deliveries met all four of these measures.

**Table 1:** **Distribution of characteristics of neonatal intensive care units (n = 84) participating in the VON days delivery room resuscitation audit**

	***N (%)***
**NICU Type**	
Ventilation restrictions or no major surgery	12 (14.6)
No ventilation restrictions; neonatal surgery except cardiac surgery	53 (64.6)
No ventilation restrictions; neonatal surgery including cardiac surgery	17 (20.7)
**International**	5 (6.0)
**Teaching**	53 (63.1)
**Nonprofit**	71 (84.5)
**Policies about Delivery Room Resuscitation**	
Deliveries that require attendance	70 (83.3)
Personnel that should attend	68 (81.0)
Training for those personnel	66 (78.6)
Briefing prior to delivery	7 (8.3)
Briefing after delivery	5 (6.0)
Family communication	8 (9.5)
**Safety checklist**	12 (14.3)
**Simulation-based neonatal resuscitation training**	68 (81.0)
**Videotape deliveries**	2 (2.4)

**Table 2:** **Distribution of infant characteristics (n = 609) audited in the VON days delivery room resuscitation audit**

	***N (%)***
**Gestational age at birth (weeks)**	
<25	17 (2.8)
25-28	61 (10)
29-32	102 (16.7)
33-37	229 (37.6)
>37	200 (32.8)
**Delivery characteristics**	
Cesarean section	381 (62.7)
Prematurity	374 (61.4)
Fetal distress	144 (23.8)
Multiple gestation	95 (15.7)
Known congenital malformation	43 (7.1)
Other	238 (39.1)
**Delivery team**	
NICU nurse	520 (85.4)
Respiratory therapist	386 (63.4)
Neonatal nurse practitioner	313 (51.4)
Attending neonatologist	286 (47.0)
Labor and delivery nurse	208 (34.2)
Neonatology fellow	54 (8.9)
Medical student, trainee	17 (2.8)
Other	211 (34.7)
**Accordance with audit objectives**	
Team composition met unit policies or guidelines	537 (88.2)
Briefing occurred before delivery	401 (65.8)
Debriefing occurred after delivery	115 (18.9)
Family communication within 30 minutes of delivery	556 (92.0)
All four objectives met	87 (14.3)

## Discussion

The purpose of this quality audit was to evaluate whether NICUs had written policies and guidelines for teamwork behaviors in neonatal resuscitation and whether those policies and guidelines were put into practice. We found that only 14% of audited deliveries met the NICU policies and guidelines for team composition, briefing, debriefing, and communication with family members following delivery. Although these results are from 2012, the results would likely be similar today.

For team composition, eight out of ten NICUs had written policies on what kinds of deliveries required resuscitation teams, who should attend such deliveries, and the training required for those team members. Overall, 88% of the audited deliveries had the appropriate team members in attendance. Identification and appropriate participation of team members did not appear to be a concern for these NICUs.

Eight percent of units had policies on briefing before delivery, yet briefing occurred before 66% of deliveries. Briefing, or sharing information before starting resuscitations, is considered a key measure of a highly-functioning team [6,9] and is recommended in the most recent international consensus guidelines on neonatal resuscitation [19]. We did not ask about the content or rigor of the briefing. We did find that only 12 NICUs (14%) used checklists, which can remind team members of their roles and responsibilities and ensure the presence of necessary equipment [24]. Use of a checklist at University of California – San Diego improved communication between resuscitation team members [25]. A checklist used in conjunction with multidisciplinary conferences, a dedicated resuscitation nurse, and frequent feedback to clinicians significantly improved delivery room care, respiratory outcomes, and length of stay for very preterm infants [26].

Debriefing was the one area where both policy documentation and practice fell short. Only five NICUs (6%) had policies stating that debriefings should occur within 24 hours of delivery, and debriefing occurred after 19% of deliveries. Debriefing is another important team function recommended in the consensus guidelines for neonatal resuscitation teams [19]. It is a mechanism for providing feedback and learning from error, and plays an important role in a continuous quality improvement environment [27,28]. While the audit asked if debriefings occurred with 24 hours of the delivery, we believe these conversations should include the entire team and should happen as soon as possible after the delivery. Some findings may require immediate action. Reviewing video recordings of simulated or actual resuscitations facilitates debriefing by providing context for feedback. Only two NICUs in this audit regularly recorded resuscitations. Recordings can assess team performance [29-31], the resuscitation environment and equipment [29,30], and fidelity with resuscitation guidelines [31-33]. One study found weekly debriefing with video review improved teamwork skills [34].

Many NICUs lacked documentation about briefing or family communication but applied these practices to deliveries. Ten percent of units had policies stating that family communication should occur within 30 minutes of delivery, but communication occurred following 92% of audited deliveries. The audit did not ask about the content or quality of the communication following resuscitation.

Multidisciplinary simulation-based team training allows learners to practice resuscitation as part of a team in a lower-stress environment than an actual resuscitation [35]. The 2010 consensus guidelines recommend using simulation in resuscitation education [19], and 82% of the NICUs in this audit reported using a simulation-based training program. NeoSim was one of the first such programs and was the first to incorporate behavioral as well as technical skills [36]. The 2010 revision of the NRP introduced simulation-based education with post-scenario debriefing to encourage teamwork training [12]. Simulation-based learning leads to better performance in simulated resuscitations than traditional learning [37-41]. Additionally, NRP trainers found simulation training increased their knowledge, skills, and confidence to train others [42].

## Conclusions

Neonatal resuscitation is a complex process that requires the coordinated action of multiple team members. Standardizing and documenting teamwork-related processes for procedures like neonatal resuscitation can increase team performance consistency and decrease the potential for error [4,5]. Among these 84 NICUs we found opportunities to improve documentation and implementation of delivery room resuscitation teamwork policies. Given the high prevalence of delivery room resuscitation, particularly for very low birth weight infants, we recommend that every NICU convene a multidisciplinary team, audit their practices, assess the data, and begin a quality improvement program to create, test, and implement standardized policies and guidelines particularly regarding briefing, debriefing, and parental communication.

## Additional file

Additional file 1:
**Hospitals participating in the VON Days Delivery Room Resuscitation Audit.**
