# Supplementary material for: Identifying improvements for delivery room resuscitation management: results from a multicenter safety audit
Source: Matern Health Neonatol Perinatol. 2015 Jan 22;1:2. doi: 10.1186/s40748-014-0006-x (PMC4772755; doi:10.1186/s40748-014-0006-x)
Supplement: Additional file 1: — Hospitals participating in the VON Days Delivery Room Resuscitation Audit. [file 40748_2014_6_MOESM1_ESM.docx]

Additional file 1. Hospitals participating in the VON Days Delivery Room Resuscitation Audit

|  | *Hospital Name* | *City* | *State* | *Country* |
| --- | --- | --- | --- | --- |
| *1* | DeVos Children's, Spectrum Health | Grand Rapids | Michigan | United States |
| *2* | Wesley Medical Center | Wichita | Kansas | United States |
| *3* | McKay Dee Hospital Center | Ogden | Utah | United States |
| *4* | Henry Zarrow Neonatal Intensive Care Unit | Tulsa | Oklahoma | United States |
| *5* | Toledo Children's Hospital | Toledo | Ohio | United States |
| *6* | Vermont Children's at Fletcher Allen Health Care | Burlington | Vermont | United States |
| *7* | Randall Children's Hospital at Legacy Emanual | Portland | Oregon | United States |
| *8* | Parkview Women's and Children's Hospital | Fort Wayne | Indiana | United States |
| *9* | MHUMC Savannah | Savannah | Georgia | United States |
| *10* | Cardinal Glennon Children's Hospital | St. Louis | Missouri | United States |
| *11* | Aultman Hospital | Canton | Ohio | United States |
| *12* | University of Tennessee Medical Center | Knoxville | Tennessee | United States |
| *13* | Presbyterian St. Luke's Medical Center | Denver | Colorado | United States |
| *14* | St. Peter's Medical Center | New Brunswick | New Jersey | United States |
| *15* | Greater Baltimore Medical Center | Baltimore | Maryland | United States |
| *16* | UMass Memorial Healthcare | Worcester | Massachusetts | United States |
| *17* | St. Luke's Regional Medical Center | Boise | Idaho | United States |
| *18* | St. Joseph's Health Center | Syracuse | New York | United States |
| *19* | Sparrow Hospital | Lansing | Michigan | United States |
| *20* | CHOI at OSF St. Francis Medical Center | Peoria | Illinois | United States |
| *21* | Albany Medical Center | Albany | New York | United States |
| *22* | Northside Hospital | Atlanta | Georgia | United States |
| *23* | Mercy Children's Hospital | Toledo | Ohio | United States |
| *24* | St. Cloud Hospital | Saint Cloud | Minnesota | United States |
| *25* | Kentucky Children's Hospital | Lexington | Kentucky | United States |
| *26* | St. Mary's Hospital Medical Center | Madison | Wisconsin | United States |
| *27* | Children's Hospital at Providence, Alaska, The | Anchorage | Alaska | United States |
| *28* | Rogue Valley Medical Center | Medford | Oregon | United States |
| *29* | Wheaton Franciscan Healthcare at St. Joseph | Milwaukee | Wisconsin | United States |
| *30* | St. Joseph Hospital and Medical Center | Paterson | New Jersey | United States |
| *31* | Christiana Care Health Services | Newark | Delaware | United States |
| *32* | Connecticut Children's Medical Center | Hartford | Connecticut | United States |
| *33* | Eastern Maine Medical Center | Bangor | Maine | United States |
| *34* | Henry Ford Hospital | Detroit | Michigan | United States |
| *35* | Scott and White Hospital | Temple | Texas | United States |
| *36* | Betty H. Cameron Women's and Children's Hospital | Wilmington | North Carolina | United States |
| *37* | Rotunda Hospital | Dublin |  | Ireland |
| *38* | Elliot Hospital | Manchester | New Hampshire | United States |
| *39* | Conemaugh Memorial Medical Center | Johnstown | Pennsylvania | United States |
| *40* | Loma Linda University Children's Hospital | Loma Linda | California | United States |
| *41* | Rockford Memorial Hospital | Rockford | Illinois | United States |
| *42* | Sharp Mary Birch Hospital for Women | San Diego | California | United States |
| *43* | Children's Hospital at Bronson | Kalamazoo | Michigan | United States |
| *44* | University Kebangsaan Malaysia | Kuala Lumpur |  | Malaysia |
| *45* | Rainbow Babies and Children's Hospital | Cleveland | Ohio | United States |
| *46* | Gundersen Lutheran Medical Center | LaCrosse | Wisconsin | United States |
| *47* | Shady Grove Adventist Hospital | Rockville | Maryland | United States |
| *48* | Christ Hospital and Medical Center | Oak Lawn | Illinois | United States |
| *49* | Medical Center at Columbus Regional, The | Columbus | Georgia | United States |
| *50* | Women's and Children's Hospital | Lafayette | Louisiana | United States |
| *51* | Hennepin County Medical Center | Minneapolis | Minnesota | United States |
| *52* | Akron Children's S.C.N. at St. Elizabeth | Youngstown | Ohio | United States |
| *53* | Baptist St. Anthony's Health System | Amarillo | Texas | United States |
| *54* | Beth Israel Deaconess Medical Center | Boston | Massachusetts | United States |
| *55* | St. Elizabeth Regional Medical Center | Lincoln | Nebraska | United States |
| *56* | North Central Baptist Hospital | San Antonio | Texas | United States |
| *57* | Southmead Hospital | Bristol | England | United Kingdom |
| *58* | Edward Hospital and Health Services | Naperville | Illinois | United States |
| *59* | St. Joseph Mercy Hospital | Ann Arbor | Michigan | United States |
| *60* | West Virginia University School of Medicine | Morgantown | West Virginia | United States |
| *61* | Ochsner Clinic Foundation | New Orleans | Louisiana | United States |
| *62* | Affinity NICU at St. Elizabeth Hospital | Appleton | Wisconsin | United States |
| *63* | Mt. Carmel Health Systems | Columbus | Ohio | United States |
| *64* | Northeast Georgia Medical Center | Gainesville | Georgia | United States |
| *65* | Connecticut Children's NICU at Uconn Health Center | Farmington | Connecticut | United States |
| *66* | Holy Redeemer Hospital and Medical Center | Meadowbrook | Pennsylvania | United States |
| *67* | Children's Memorial Hermann Hospital | Houston | Texas | United States |
| *68* | Texas Health Presbyterian Hospital Plano | Plano | Texas | United States |
| *69* | Washington Hospital Center | Washington | District of Columbia | United States |
| *70* | Adventist Hinsdale Hospital | Hinsdale | Illinois | United States |
| *71* | St. Alexius Medical Center | Hoffman Estates | Illinois | United States |
| *72* | Centra Health, Virginia Baptist Hospital | Lynchburg | Virginia | United States |
| *73* | Woman's Hospital of Texas,The | Houston | Texas | United States |
| *74* | Stony Brook University Medical Center | Stony Brook | New York | United States |
| *75* | Steven & Alexandra Cohen Children's Med.Cen. Of NY | New Hyde Park | New York | United States |
| *76* | Carilion Clinic Children's Hospital | Roanoke | Virginia | United States |
| *77* | William Beaumont Hospital | Royal Oak | Michigan | United States |
| *78* | Patologia Neonatale Ospedale San Bortolo | Vicenza |  | Italy |
| *79* | CoxHealth Neonatology | Springfield | Missouri | United States |
| *80* | Neocenter S.A. | Belo Horizonte |  | Brazil |
| *81* | Hospital of Central Connecticut, The | New Britain | Connecticut | United States |
| *82* | Kapiolani Medical Center for Women and Children | Honolulu | Hawaii | United States |
| *83* | University of Texas Southwestern Med. Ctr. Dallas | Dallas | Texas | United States |
| *84* | St. Luke's Baptist Hospital | San Antonio | Texas | United States |
